# Positive Correlation between Enhanced Expression of TLR4/MyD88/NF-κB with Insulin Resistance in Placentae of Gestational Diabetes Mellitus

**DOI:** 10.1371/journal.pone.0157185

**Published:** 2016-06-24

**Authors:** Hui Feng, Rina Su, Yilin Song, Chen Wang, Li Lin, Jingmei Ma, Huixia Yang

**Affiliations:** Department of Obstetrics and Gynecology, Peking University First Hospital, Beijing, China; Virgen Macarena University Hospital, School of Medicine, University of Seville, SPAIN

## Abstract

Insulin resistance (IR) is a critical factor of the pathophysiology of Gestational diabetes mellitus (GDM). Studies on key organs involved in IR, such as livers and adipose tissues, showed that Toll-like receptor 4 (TLR4) can regulate insulin sensitivity. As a maternal-fetal interface with multi-functions, placentae could contribute to the development of IR for GDM. Thus, we investigated the expressions of TLR4/Myeloid Differentiation factor 88 (MyD88)/Nuclear Factor kappa-light-chain-enhancer of activated B cells (NF-kB) in term placentae from 33 GDM women and 36 healthy pregnant women with normal glucose tolerance, evaluated local and systemic IR and furthermore identified the association between placental TLR4 and IR. TLR4 protein was expressed in various cells of term placenta, particularly in syncytiotrophoblast of villi. Compared with normal pregnancy, the expression of TLR4/MyD88/NF-kB pathway increased in the placenta of GDM (*p*<0.05), and these differences were more pronounced in the maternal section of the placenta and the syncytiotrophoblast of villi. In addition, more severe IR was observed in the placenta of GDM patients than the control group, evidenced with higher pIRS-1(ser312) (*p*<0.001) and lower IRS-1 (*p*<0.05) as well as pAkt proteins (*p*<0.01). The expression of TLR4 in placentae is positively correlated with local IR (pIRS-1: r = 0.76, *p* <0.001 and *p*Akt: r = -0.47, *p* <0.001) and maternal fasting (r = 0.42, *p* <0.01), one-hour (r = 0.52, *p* <0.01) and two-hour glucose (r = 0.54, *p* <0.01) at OGTT. We found an that enhanced expression of the TLR4-MyD88-NF-kB pathway occurs in GDM placentae, which positively correlates with heightened local IR in placentae and higher maternal hyperglycemia. The TLR4/MyD88/NF-kB pathway may play a potential role in the development of IR in placentae of GDM.

## 1. Introduction

Gestational diabetes mellitus (GDM) is presented with systemic and local insulin resistance (IR). Maternal IR is a normal part of human pregnancy, particularly during the second trimester and third trimester of human pregnancy. However, in women diagnosed with GDM, IR is more pronounced. During the past decades, the underlying mechanism of IR has being investigated. Several possible candidates have been identified, including metabolic factors [1–3], mitochondrial function [4], oxidative stress [5, 6] and adipocytokines [7]. Moreover, systemic and local inflammation is thought to play an important role in the development of IR [8, 9].

While previous studies of IR concentrated mainly on livers, adipose tissues or skeletal muscles, we focused on the placenta. Placentae as a maternal-fetal interface perform various functions, and play a critical role in the development and progression of GDM. Placental-derived hormones, human placental lactogen (hPL) [10] and human placental growth hormone (hPGH) [11, 12], are believed to be a major factor in reprogramming maternal physiology to achieve an insulin-resistant state. Moreover, the up-regulated genes for inflammatory pathways in placentae of women with GDM, as well as genes for lipids, amino acid and glucose transport [13, 14], may also function in systemic or local IR.

Toll-like receptor 4 (TLR4) is a members of the TLR family of receptors that recognize pathogen-associated molecular patterns (PAMPs) as well as endogenously derived indicators of tissue injury [15]. TLR4 is a key mediator of pro-inflammatory responses. The activation of TLR4 signaling pathway leads to the generation of pro-inflammatory cytokines through the up-regulation of several transcription factors, including NF-κB, activated protein 1 (AP-1), and interferon regulatory factors (IRFs) [16]. These cytokines can inhibit insulin action in insulin target cells, directly or indirectly (reviewed in [8]).

TLR4 is a potential molecule function in IR. Enhanced expression of TLR4 has been observed in animal models of diabetes and in T1DM and T2DM patients [17–19]. Our previous studies have also found the expression of TLR4 in PBMC, skeletal muscles and adipose tissues was distinctively increased in GDM patients. Furthermore, blocking TLR4 signaling can improve IR. Jia et al. found hepatocyte-specific TLR4 deficiency attenuates diet-induced whole-body and local IR, mainly in the liver and adipose tissue [20].

The expression of TLR4, as well as its various co-receptors and necessary proteins, including CD14, has been described in human placentae [21, 22], and seems to be altered in pregnancy complications. Kumazaki et al. found TLR4 expression in the villous Hofbauer cells was higher in preterm placentae with chorioamnionitis (CAM) than in preterm placentae without CAM, or term placentae with or without CAM [23]. Li et al. observed higher TLR4 levels occur in placentae of pregnancies complicated with early-onset preeclampsia [24]. However, TLR4 expression in placentae of GDM is unknown. Thus, we hypothesized that the expression of TLR4/MyD88/NF-kB pathway will alter in placentae of GDM. Furthermore, given the close association between TLR4 and IR in livers and adipose tissues, and limited data in tissue-specific IR in placenta, we assessed local IR in placentae of GDM and identified its association with TLR4/MyD88/NF-kB pathway in placentae.

## 2. Materials and Methods

### 2.1. Patients and tissue samples

Human placental samples and medical records were collected from normal and GDM term pregnancies after elective caesarean section in Peking University First Hospital during Devember 2014 to Junn 2015. Eligibility criteria included all women delivering in the sample period, who were carrying a singleton fetus, and that have performed a 75g OGTT between 24 to 28 weeks of gestation. We excluded women who delivered prior to 28 weeks of gestation, those with either known pre-pregnancy diabetes mellitus or overt diabetes diagnosed during pregnancy. Venous blood sample collected after at least 8 hours of fasting. Glucose values above 7.0mmol/L or 11.1mmol/L, at fasting and 2-h post glucose load, respectively, were considered as overt diabetes and were excluded from the study. Excluding diabetes women, a patient will be considered GDM when any one of the following criteria is met or exceeded in the OGTT: 0 h (fasting) ≥5.10mmol/L; 1 h ≥10.00mmol/L; and 2 h ≥8.50mmol/L [25]. Patients were excluded if they had DM, multi-fetal gestation and any other complications, including Preeclampsia (PE), polycystic ovarian syndrome (PCOS), preterm delivery and intrauterine infection. The GDM patients were matched for maternal age, BMI before pregnancy, at first- trimester and at delivery, as well as gestational age at delivery (within 2 weeks) with the control. The information had been identifying after data collection. All patients provided written informed consent before the collection of placental biopsy samples. The collection and use of samples in this study were approved by the Ethics Committee of Peking University First Hospital.

Both maternal and fetal sections of placental tissue were collected from 33 GDM patients and 36 non-diabetic patients. Placental tissues were taken within 15 min of operative delivery, immediately snap-frozen in liquid nitrogen and then stored at −80°C.

### 2.2. Immunohistochemistry

Immunohistochemistry staining was performed on sections of Formalin-Fixed and Parrffin-Embedded (FFPE) placental specimens of both maternal and fetal surfaces from GDM patients and normal pregnancies. Membranes were blocked for 1h and subsequently incubated with antibodies against TLR4, MyD88, NF-κB(p65), pIRS-1(ser312) and pAkt(ser 473) (all from Abcam, USA) overnight at 4°C and with horseradish peroxidase-conjugated secondary antibodies (Zhongshan Goldernbridge Biotechnology Co., Beijing, China) for 60 min at room temperature. Negative controls consisted of sections exposed to 2% Bovine Serum Albumin (BSA) and phosphate buffer saline (PBS) instead of primary antibody. Membranes were visualized using a 3, 3'-diaminobenzidine (DAB) kit and counterstained with hematoxylin (Zhongshan Goldernbridge Biotechnology Co., Beijing, China). Inaddition, a mouse monoclonal antibody against cytokeratin 7 (CK7) and a mouse monoclonal anti-human Vimentin (V9) antibody (from Abcam, USA) were used for identified trophoblast cellesand decidual cells, respecitively. Results were assessed based upon the evaluations of three independent observers. All slides were scanned using an Olympus IX71 microscope equipped with a DP72 digital camera system, and was analyzed with the Image-pro plus-Version 6 software.

### 2.3. RNA isolation and real-time PCR

Total RNA was isolated from placental specimens using TRIzol (ambion, CA) reagent. cDNA was synthesized using a High Capacity cDNA Reverse Transcription Kit (Applied Biosystems, Life Technologies, USA) according to the manufacturer’s recommendations. The cDNA expression level was quantified by a real time PCR with SYBR Green selected PCR Master Mix (Applied Biosystems, Life Technologies, USA) according to the manufacturer’s protocol. Real-time PCR was performed with the ABI PRISM 7500 Sequence Detector System (Applied Biosystems, Life Technologies, USA). The primers sequences are depicted below (Table 1). All reactions were carried out in triplicate. Statistical analysis of the results was performed with the △Ct value (Ct gene of interest–Ct GAPDH). The △△CT method of relative quantification was used to determine the fold change in the expressions [26].

**Table 1:** The sequence of the primer for PCR.

Gene	Forward primer	Reverse primer
**TLR4**	5’-CTGCAGGTGCTGGATTTATCC-3’	5’-GGTGGCTTAGGCTCTGATATGC-3’
**MyD88**	5’-ACTGCTCGAGCTGCTTACCAA-3’	5’-CTCCTGCTGCTGCTTCAAGAT-3’
**NF-κB**	5’-CCCATCTTTGACAATCGTGC-3’	5’-CTGGTCCCGTGAAATACACC-3’
**GAPDH**	5’-GAAGGTGAAGGTCGGAGTC-3’	5’-GAAGATGGTGATGGGATTTC-3’

### 2.4. Western Blotting

Placental tissues were extracted and western blotting was performed as previously described [24]. Soluble cytoplasmic proteins were extracted by incubating the homogenized placental tissues with lysis buffer (RIPA and 100 mM Phenylmethylsulfonyl fluoride [PMSF]) (Sigma-Aldrich, St. Louis, MO, USA), and the concentration was measured with bicinchoninic acid protein assay. 50μg total protein extract was subjected to 10% Sodium Dodecyl Sulfate Polyacrylamide Gel Electrophoresis (SDS-PAGE) and transferred to a nitrocellulose membrane (Amersham Pharmacia Biotech, Buckinghamshire, UK). After blocking with 5% skim milk or BSA in phosphate-buffered saline (PBS) with 0.1% Tween-20(TBST) for 1 h at room temperature, the membranes were probed with primary antibodies directed against human TLR4, MyD88, IRS-1, phospho-IRS-1 (Ser312), Akt, phospho-Akt (Abcam, USA) and phospho-NF-kB p65(ser536) (Santa Cruz Biotechnology) overnight at 4°C. The membranes were then incubated with horseradish peroxidase streptavidin-linked second antibodies (Zhongshan Goldernbridge Biotechnology Co., Beijing, China) for 1 h at room temperature and exposed to enhanced chemiluminescence reagent (ECL; Millipore, USA). Western blot data were standardized against Glyceraldehyde 3-phosphate dehydrogenase (GAPDH), and the relative density was analyzed with the Quantity One software (Bio-Rad Laboratories Pty., Australia) and presented as mean ± standard deviation. Western blots were performed three times for the quantification.

### 2.5. Statistical Analyses

All data were analyzed with the SPSS 20.0 software (Chicago, USA). Differences between groups were analyzed using independent t-test or rank-sum test depending on the variable characteristic. Pearson’s and Spearman’s correlation test ware used to study the association between the expression of TLR4 and IR or maternal glucose at OGTT. Differences with *p*-values below 0.05 were considered significant

## 3. Results

### 3.1. The baseline characteristic of patients

During the study period, placental tissues of 69 maternal sections and the same fetal sections were collected from 33 GDM patients and 36 normal pregnant women. The baseline characteristics were shown in Table 2. There were no differences between the GDM group in terms of maternal age, BMI at first-trimester and at birth, gestational age at operative delivery relative to the values obtained for the control group. Moreover, fasting, one-hour and two-hour plasma glucose concentrations at OGTT were significantly higher in the GDM participants compared with the control group.

**Table 2:** Clinical characteristics.

	Control(n = 36)	GDM(n = 33)	*p-value*
**Maternal age (years)**	30.53 ±3.10	31.24 ±2.68	0.31
**Maternal BMI at first trimester (kg/m**^**2**^**)**	23.93 ±3.81	25.73 ±4.58	0.08
**Maternal BMI at birth (kg/m**^**2**^**)**	29.05 ±3.79	29.96 ±4.45	0.37
**Gestational age at delivery (weeks)**	39.38 ±0.95	39.52 ±0.88	0.14
**Fasting plasma glucose (mmol/L)**	4.64 ±0.36	5.51 ±1.33	<0.001
**OGTT-1h (mmol/L)**	7.54 ±1.21	9.93 ±1.74	<0.001
**OGTT-2h (mmol/L)**	6.40 ±0.93	8.27 ±1.71	<0.001

GDM: gestational diabetes metillus; BMI: body mass index; OGTT: oral glucose tolerance test.

### 3.2. The distribution of TLR4 in normal term Placentae

**Fig 1:**
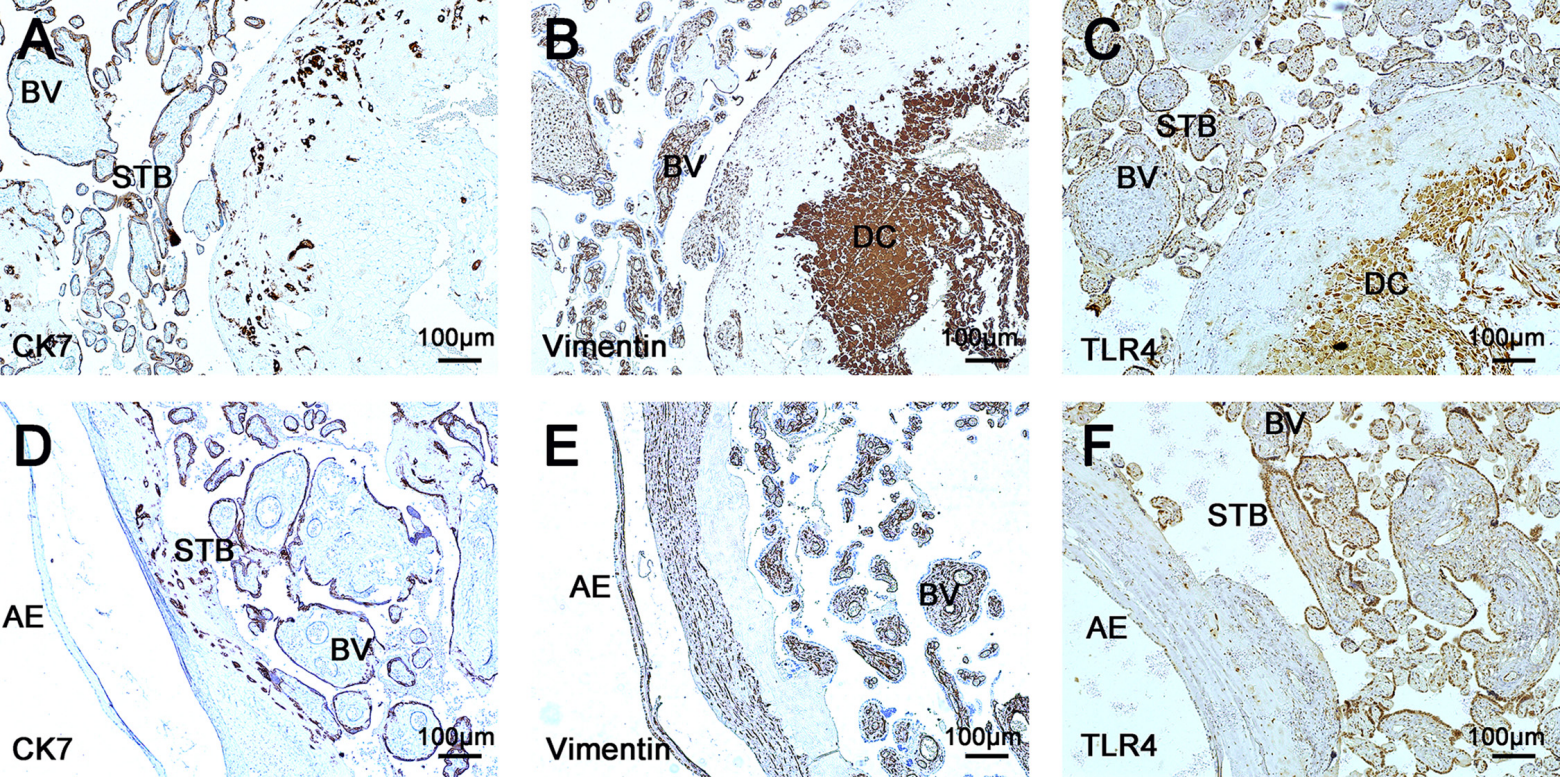
Immunostaining for TLR4 in normal term placentae. (A) The expression of CK7 in the maternal surfaces of placentae; (B) The expression of vimentin in the maternal surfaces of placentae; (C) The expression of TLR4 in the maternal surfaces of placentae. In the serial sections of maternal surfaces of normal placentae, TLR4 expressions were observed in trophoblast, decidual cells and in the vascular endothelial cells (n = 36). (D) In the fetal sections of placentae, the straining of CK7 were observed; (E) The expression of vimentin in fetal section of normal placentae (n = 36); (F) The expression of TLR4 in the fetal surface of placentae. Except in trophoblasts and vascular endothelial cells, TLR4 staining were also observed in amniotic epithelium (n = 36). STB, syncytiotrophoblast; BV: blood vascul; DC, decidual cells; AE, amniotic epithelium. CK7: cytokeratin 7; TLR4: toll like receptor 4. Positive staining is brown. Magnification ×100.

Firstly, we characterized the distribution of TLR4 in normal term placentae. As shown in Fig 1, TLR4 is widely expressed in human term placental tissues, in both maternal and fetal sections. Using the specific marker, CK7 (Fig 1A & 1D) and Vimentin (Fig 1B & 1E), for trophoblast and decidual cell respectively. TLR4 are identified in trophoblast cell and decidual cells (Fig 1C & 1F). It is expressed strongly both in the trophoblasts covering the peripheral chorionic villi and decidual cells (Fig 1C). Moreover, TLR4 is also expressed in vascular endothelial cells and amniotic epithelium (Fig 1F).

### 3.3. The expression of TLR4/MyD88 in chorionic villi of GDM Placentae

**Fig 2:**
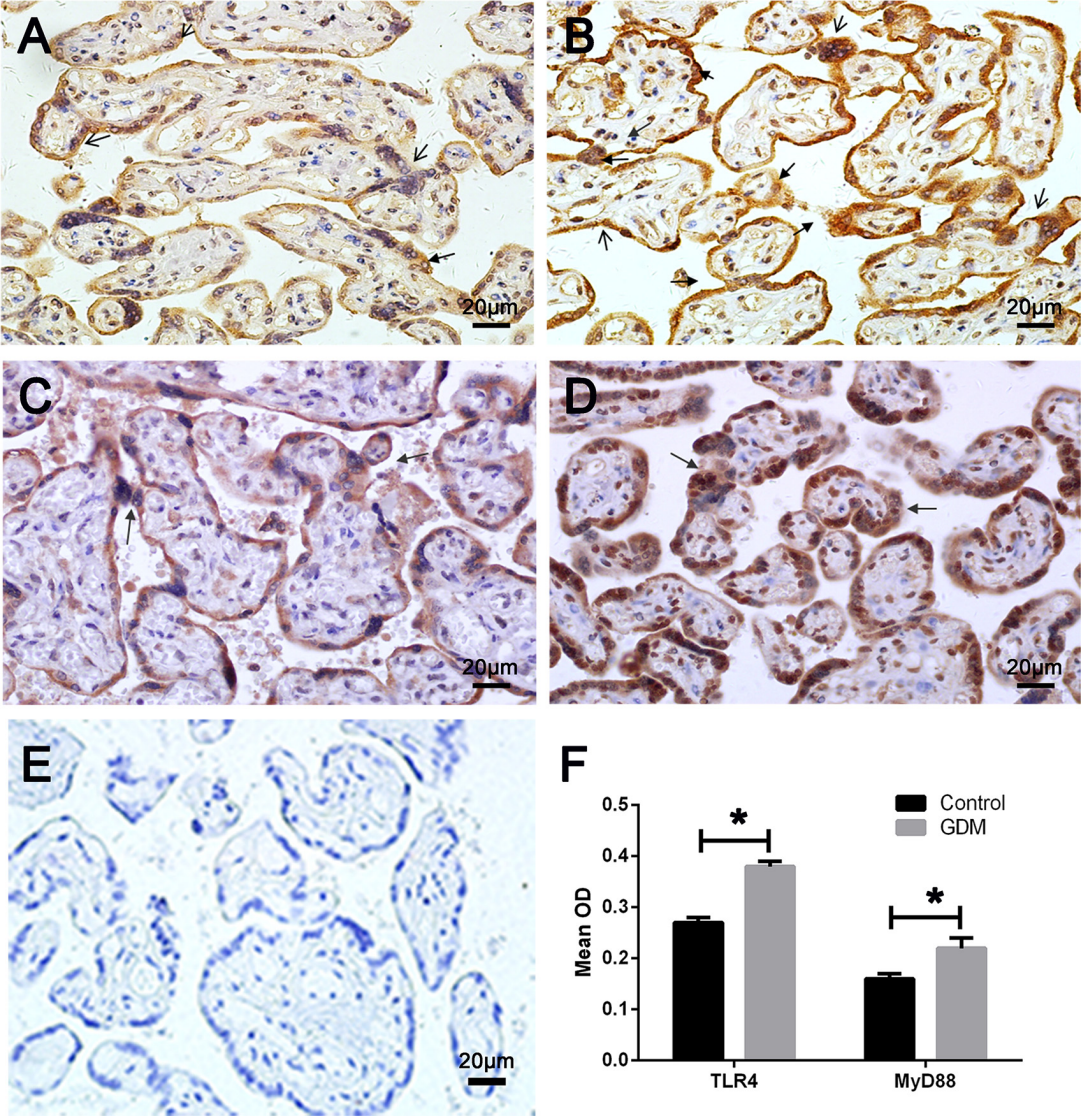
Immunohistochemistry of TLR4 and MyD88 in chorionic villi of normal control (n = 36) and GDM (n = 33). TLR4 protein was expressed in chorionic villi of (A) normal and (B) GDM placentae, a more distinct staining was observed in the trophoblast cell of GDM than the control term placentae (GDM: 0.38±0.01 vs control: 0.27±0.01, p = 0.02). The staining for MyD88 was detected in (C) the normal placentae and (D) the GDM placentae. (E) Negative control. (F) The comparation of TLR4 and MyD88 in placentae between control and GDM. Compared with the control group, enhanced MyD88 staining was shown in GDM (GDM: 0.22±0.02 vs control: 0. 16±0.01, p = 0.03). Sections were counterstained with hematoxylin (blue). The arrows indicate positive cells. All photomicrographs were taken at 400×magnification.

Immunohistochemistry was used to detect the expressions of TLR4 and its downstream molecule MyD88 in placentae of GDM. As shown in Fig 2, staining for TLR4 was mainly observed in trophblast cells and vascular endothelial cells of chorionic villi. Compared with normal placentae (Fig 2A), TLR4 staining was significantly enhanced in GDM placentae (Fig 2B & 2F). The expression of MyD88 showed similar characteristics, with a more obvious staining in GDM (Fig 2D & 2F) than the control group (Fig 2C).

### 3.4. The expression of TLR4/MyD88/NF-kB pathway in maternal and fetal surfaces of GDM Placentae

**Fig 3:**
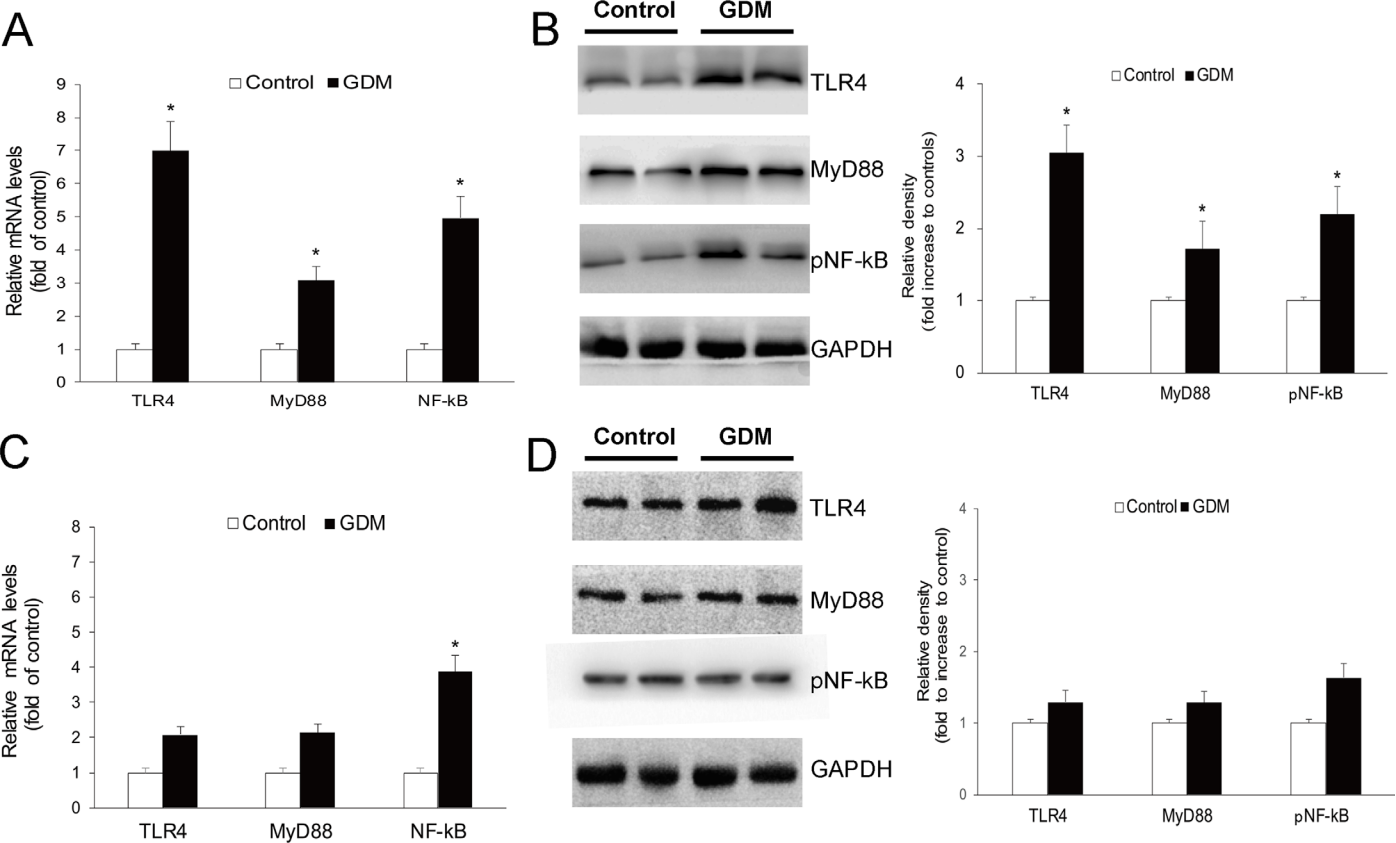
Expression of the TLR4/MyD88/NF-kB pathway in maternal and fatal sections of normal gestation (n = 36) and GDM placentae (n = 33). Expression of TLR4, MyD88 and NF-kB (A)mRNA and (B) protein in the maternal surfaces of placentae, which showed significantly increases in GDM placentae than the control. And these expression as (C) mRNA and (D) protein in the fetal section of placentae showed a similar trends but not reached significantly differences.

The content of key molecules of the TLR4 pathway was quantified by real-time PCR and western blot. Fig 3A shows TLR4, MyD88, and NF-kB mRNA levels in the maternal section of placentae. In comparison with the normal group, mRNA was markedly overexpressed (about 7-fold increase) for TLR4 in the placentae of GDM patients, while a lesser extent for MyD88 and NF-kB, approximately 3-fold and 4-fold increase was observed, respectively. The protein expression of TLR4 and its downstream molecules on placental maternal surfaces is shown in Fig 3B. Consistent with the expression of mRNA, significantly higher levels of TLR4, MyD88 and NF-kB (p65) proteins were observed in GDM maternal placentae (*p*<0.05).

The expression of the TLR4/MyD88/NF-kB pathway in fetal surfaces of placenta was detected also. In accordance with those expressions on maternal sections, we observed increased trends of TLR4, MyD88 and NF-kB for fetal sections of GDM placentae than the control, both expressed as mRNA (Fig 3C) and proteins (Fig 3D). Furthermore, compared with maternal sections, these expression gaps of the TLR4/MyD88/NF-kB pathway between the GDM and normal control seem less obvious with no significant difference in the fatal surfaces of the placentae.

### 3.5. The local IR in the placenta of GDM patients

**Fig 4:**
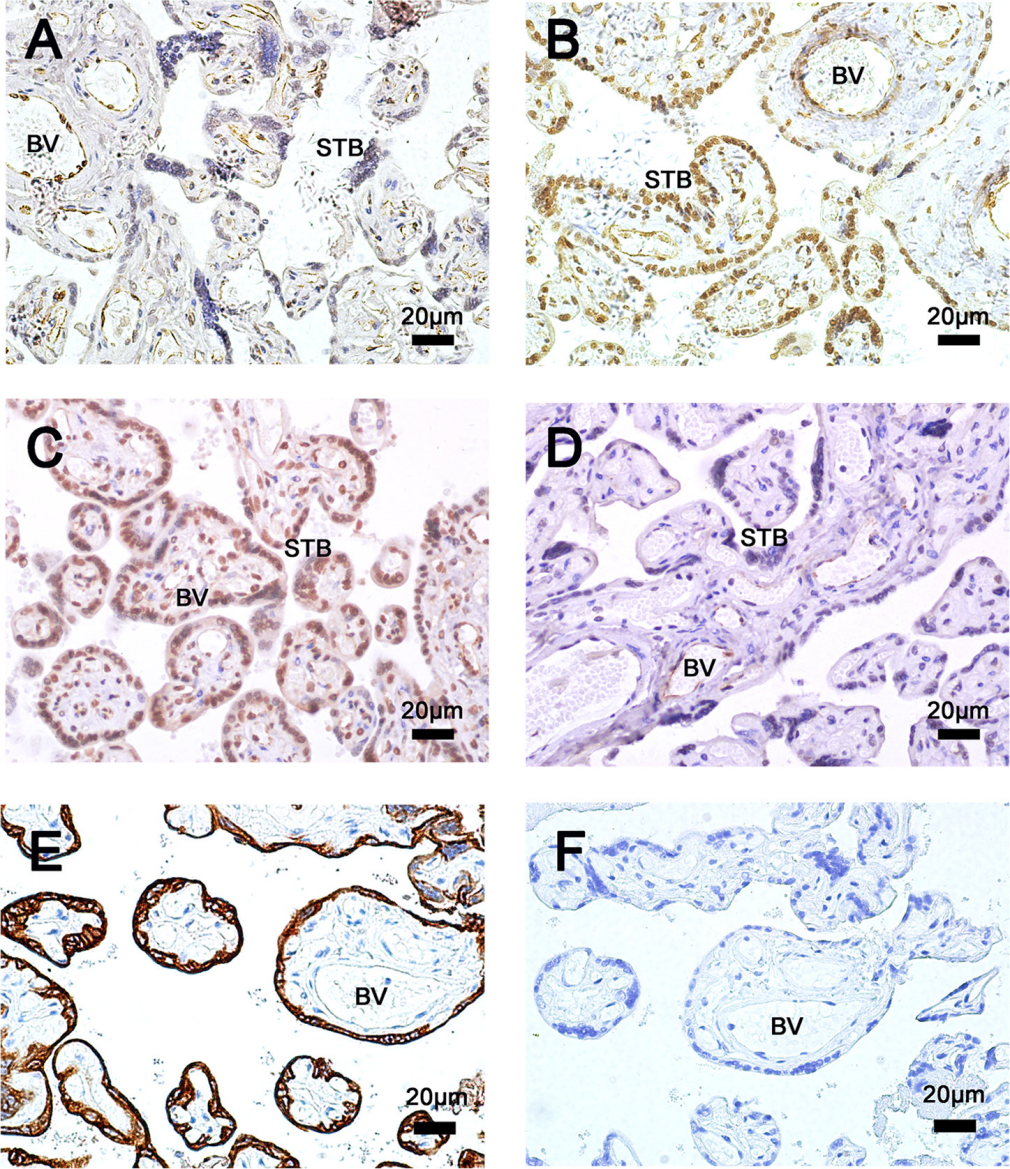
Immunohistochemistry of pIRS-1(Ser312) and pAKt in chorionic villi in maternal surface of normal control (n = 36) and GDM (n = 33). Protein of pIRS-1(Ser312) were expressed in trophoblast and endothelial cells of (A) the normal, and its expression increased in (B) GDM placentae. Phosphorylation of Akt protein was strongly expressed in (C) normal placentae, whereas it was absent or very faintly expressed in (D) GDM placentae. (E) The expression of CK7 in chorionic villi in maternal surface of placentae. (F) Negative control. The decrease mainly happened in trophoblasts (CK7+cells) but not endothelial cells of vessel. Sections were counterstained with hematoxylin (blue). All photomicrographs were taken at 400×magnification. STB, syncytiotrophoblast; BV: blood vascul.

To investigate whether local insulin resistance of placentae alters for GDM patients, we examined the key molecules in the insulin pathway. Compared to the control (Fig 4A), pIRS-1(Ser312) staining in the placentae of GDM patients (Fig 4B) was significantly increased (mean OD: 0.034±0.005 vs 0.083±0.009, *p*<0.05), while the phosphorylation of Akt was significantly decreased (as control in Fig 4C & GDM in 4D) (mean OD: 0.022±0.007 vs 0.009±0.002, *p*<0.05). Moreover, these changes mainly happened in trophoblasts of chorionic villi (CK7+), as the increase of pIRS-1(Ser312) was shown in trophoblast; meanwhile, the signal of pAkt was obviously absent or minimal in trophoblasts of the GDM placentae, while showed no significant difference in vessel endothelial cells.

**Fig 5:**
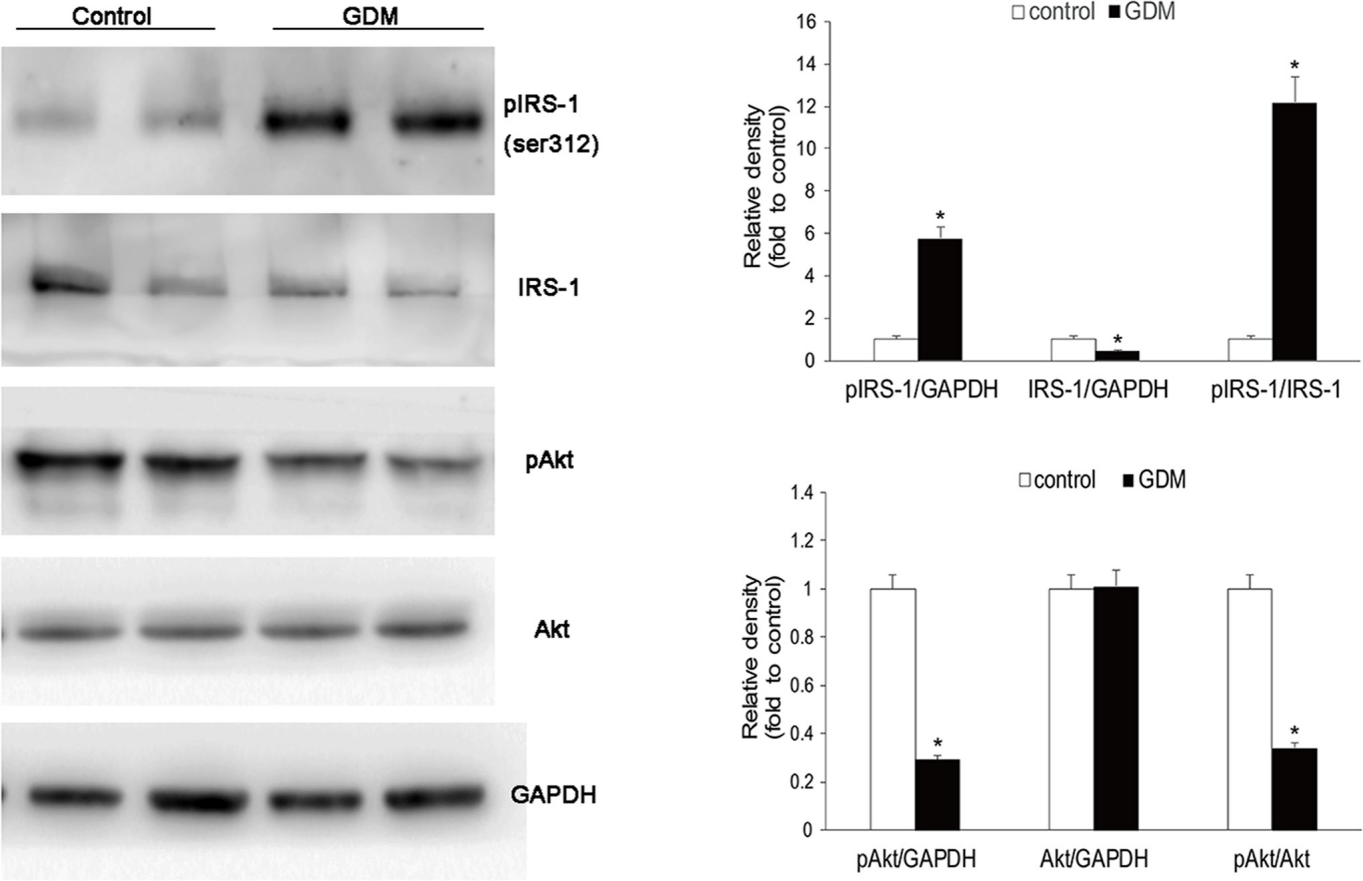
Insulin signaling pathway in placentae of GDM. Western blotting for the phosphorylation of IRS-1 (Ser312) and phosphorylation of Akt in maternal surface of placentae. Representative western blot images are shown. Compared with normal pregnancy (n = 36), the GDM placentae (n = 33) showed increased IRS-1 phosphorylation (Ser312), decreased IRS-1 and Akt phosphorylation.

Using western blot analysis, we further quantified the changes of insulin sensitivity for the GDM placentae. Compared with normal pregnancy, the GDM placentae showed increased IRS-1 phosphorylation (Ser312), decreased IRS-1 and Akt phosphorylation (Fig 5). Furthermore, increased pIRS-1/IRS-1 ratio and decreased pAkt/Akt ratio were observed in the GDM placentae. The changes of IRS-1 and Akt phosphorylation suggested a worse local IR in the GDM placentae.

### 3.6. The association between TLR4 and local IR in placentae

**Fig 6:**
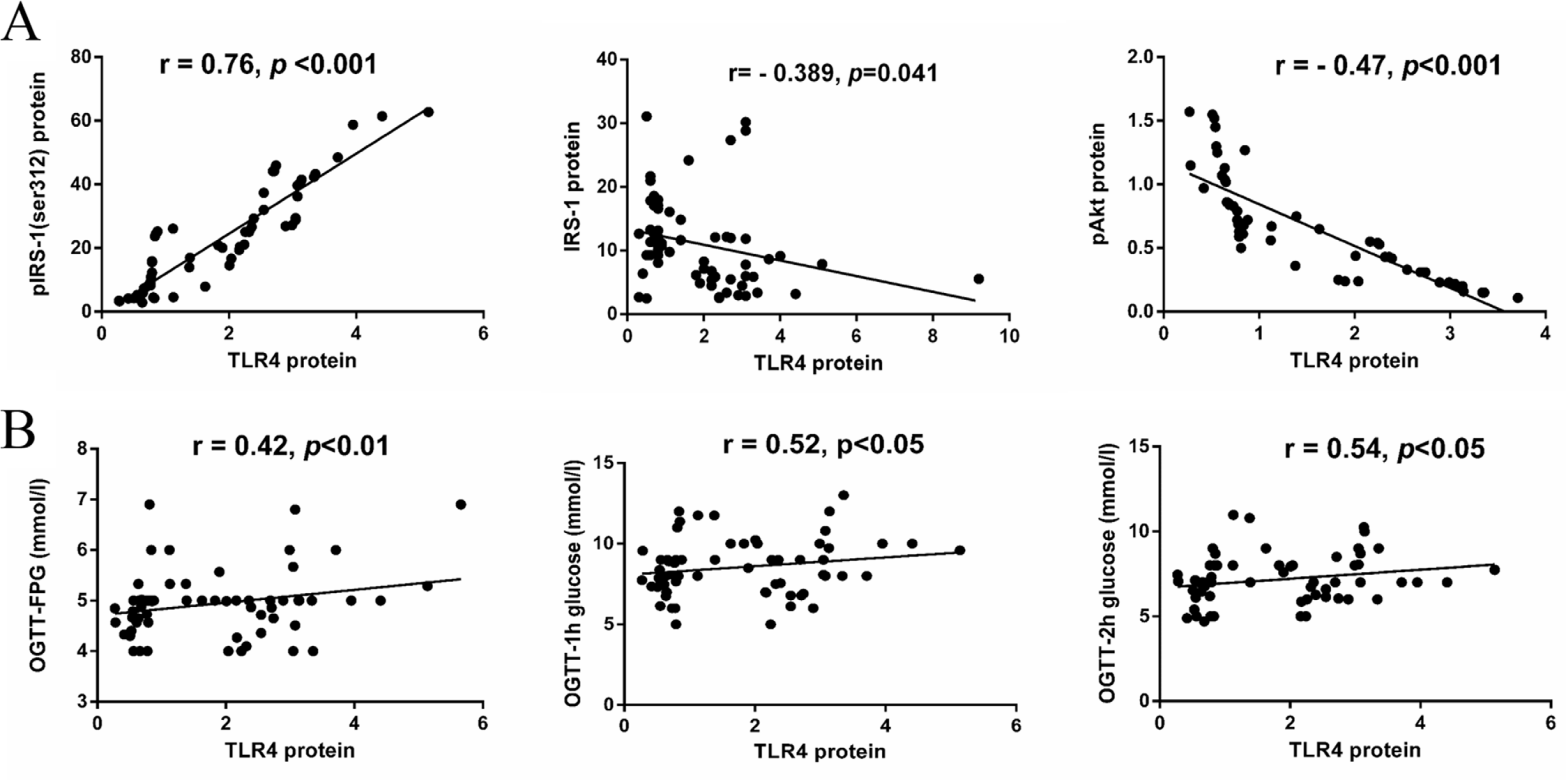
The relationship between expression of TLR4 and placental and systemic IR. **(A) The association between TLR4 and local IR in placentae.** The expression of TLR4 protein is positively correlated with the expression of pIRS-1(r = 0.76, *p*<0.001), while it’s negatively correlated with the expression of IRS-1(r = -0.26, *p*<0.05) and pAkt (r = -0.47, *p*<0.01). **(B) The correlation between placental TLR4 and maternal glucose levels.** The expression of TLR4 in placentae positively correlated with the maternal plasma glucose concentration at OGTT (fasting glucose: r = 0.42, *p* < 0.01; OGTT-1h: r = 0.52, *p* < 0.05 and OGTT-2h: r = 0.54, *p* < 0.05).

The relationship between the expression of TLR4 and local insulin signaling pathway in the placenta was analyzed (Fig 6A). The expression of TLR4 protein is positively correlated with the expression of pIRS-1(r = 0.76, *p*<0.001), while it’s negatively correlated with the expression of IRS-1(r = -0.26, *p*<0.05) and pAkt (r = -0.47, *p*<0.01).

### 3.7. The correlation between placental TLR4 and maternal plasma glucose at OGTT

We assessed the correlation between placental TLR4 and maternal glucose levels, and found the expression of TLR4 in placentae positively correlated with the maternal plasma glucose concentration at OGTT (Fig 6B). Increased TLR4 in placentae complied with higher fasting glucose (r = 0.42, *p*<0.01), high one-hour (r = 0.52, *p*<0.05) and two-hour (r = 0.54, *p*<0.05) plasma glucose concentration at OGTT.

## 4. Discussions

Although the underlying mechanisms remain to be studied, growing evidence suggests that systemic and local production/action of inflammatory molecules may be particularly critical in causing IR in GDM, including CRP [27], TNF-α [28, 29] and IL-6 [30, 31]. Several inflammatory pathways participated in local IR. The TLR4 pathway is a possible candidate which can induce a potential pro-inflammatory reaction. Upon being stimulated by LPS and adverse metabolic products, it will activate the downstream molecular MyD88, induce the translocation of transcription factor NF-kB to nuclear and trigger the production of pro-inflammatory cytokines and chemokines, including IL-6, TNF-α, chemokine (C-C motif) ligand 2 (CCL2), which may function in IR.

First of all, consistent with previous research [21, 22], the expression of TLR4 was observed in various cells of placentae, including syncytiophoblast, endothelium cell, decidual cell and amnion epithelium. However, the expression is not constant and seems to be regulated in a spatial manner. Ma et al. evaluated the expression of TLR4 in third-trimester placentae and observed most prominent staining in syncytiotrophoblast and fibroblast [32]. In this study, TLR4 was expressed both strongly and consistently in the syncytiotrophoblasts, particularly in the maternal surface.

Moreover, the expression of TLR4 and its pathway molecules changed dramatically in GDM placenta. Our data identified the enhanced expression of TLR4 and its well-recognized downward molecules, MyD88 and NF-kB, in the placentae of GDM compared with the control group. In addition, the expression of TLR4 may be associated with the location in placentae, as the expression gap of key molecules of TLR4 pathway between GDM and control placentae were more obvious for the maternal surface than the fetal surface. This may partially account for more communication with maternal blood for the maternal section of placentae. Maternal blood containing lots of irritants can induced the activation of the TLR4 pathway. Exception microbial products, endogenous signals of tissue injury can also activate TLR4, which include debris from apoptotic, damage-associated molecular patterns, ECM degradation products, high mobility group box-1 protein (HMGB1), fatty acids, heat shock proteins, AGEs) released during tissue damage and inflammation [33, 34]. Lipopolysaccharide (LPS), the major cell wall component of Gram-negative bacteria, is a key ligand of TLR4. Increased LPS has been proved on diabetes patients due to the imbalance of intestinal flora [35]. Moreover, dysfunctional lipid metabolism and the accumulation of free saturated fatty acids may also have a potential role in the activation of placental TLR4 pathway [13]. Furthermore, as the maternal and fetal compartments are in contact with different surfaces of the placenta: the microvillous membrance of the syncytiotrophoblast is in contract with the maternal blood, whereas the endothelium of the placental vasculature is in contact with the fetal blood. Thus, in the local chorionic villi of placentae, the increased expression of TLR4 and MyD88 mainly occurred in trophoblasts covered around villi.

Based mainly on the research in hepatocyte, adipose tissue and skeletal muscles, the TLR4 pathway has been proven to play an important role in local insulin sensitivity [20, 36]. In placenta, the association has been identified in our study. Impaired insulin signaling, that may cause worse IR, were detected in the placentae of the GDM. The placenta is a rich source of insulin receptor and serve as a target tissue of insulin in gestation. On one hand, the amount of insulin receptors can be regulated by ambient insulin concentrations. Hence, it comes as no surprise that in GDM insulin receptor protein is changed in total placenta and on trophoblast membranes [37, 38]. On the other hand, IR emerges from elevated levels of cytokines and hormones counter-acting insulin signaling and form altered expression levels of components of the insulin signaling cascade [39]. The insulin receptor substrate1 (IRS-1) and Akt (or PKB) are the critical nodes in the insulin phosphatidylinositol 3-kinase (PI3K) signaling pathway, which is mainly responsible for insulin-mediated metabolic effects, such as glucose uptake, glycogen synthesis and regulation of protein translation initiation. Phosphorylation at serine residues on IRS-1 protein dampens the insulin signaling cascade has been well documented [40, 41]. Increased serine phosphorylation of IRS-1 has been observed in skeletal muscles from mice and humans with GDM [42–44]. Elevated serine phosphorylation reduces insulin-stimulated insulin receptor, IRS-1tyrosine phosphorylation and PI3K activities and is accompanied by impaired glucose uptake. We found increased serine phosphorylation of IRS-1 in GDM placentae compared with normal pregnancy subjects. Meanwhile, a reduced IRS-1 protein was observed in GDM placentae, which can be account for the degradation role of serine phosphorylation IRS-1 on IRS-1protein by the proteosomal degradation pathway [45]. In addition, Akt is a serine/threonine kinase that signals most of insulin’s metabolic actions downstream of PI3K. In insulin signaling, Akt phosphorylation permit GLUT4 translocation to plasma membrane. We measured the expression levels of Akt in placentae. A declined phosphorylation of Akt, but not total Akt, was observed in placentae of GDM. In accordance to the increase of IRS-1phosphorylation on serine, these changes of the components of the insulin signaling pathway strongly suggested a state of worse IR occured in placentae of the GDM patients.

Furthermore, TLR4 had positive correlation with the local IR of placentae. The expression of TLR4 is positively correlated with the level of pIRS-1 protein and negatively associated with pAkt protein. The dysfunction of insulin signaling will affect the normal insulin sensitivity and result the insulin resistance. Thus it suggested a positive correlation between the expression of TLR4 and local IR in placentae. Moreover, increased pIRS-1 and declined phosphorylation of pAkt were detected in trophoblastes, which indicated that IR occurred mainly in trophoblastes. The latter was where enhanced staining of TLR4/MyD88 was observed, but not endothelium of vessels. Enhancing the expression and activation of TLR4/MyD88/NF-kB pathway in the placentae may increase the generation and release of inflammatory cytokines and bio-mediator, which will affect the insulin signaling and result in a significant deterioration of insulin sensitivity in GDM placenta. In addition, the expression of TLR4 in placentae was positively correlated with maternal fasting and one-hour and two-hour plasma glucose concentrations at OGTT. Increased TLR4 level in placentae was correlated with higher maternal hyperglycemia, which partly reflected a heightened whole body IR. The enhanced expression of TLR4 in placentae may also correlate with systemic IR in GDM patients. However, more investigations are required to uncover the effect of the TLR4/MyD88/NF-kB pathway on systemic and local insulin signaling and/or conversely, the effects of insulin on the TLR4/MyD88/NF-kB pathway in placentae. One limitation in this observational study is that, most of the GDM patients received intervention once diagnosed, including diet management, exercise and even insulin treatments. The influence of these treatments on the correlation is unclear and should be considered in future studies.

## 5. Conclusions

Overall, the findings of this study showed that TLR4 was expressed by various cells of term placentae, particularly in syncytiotrophoblast of villi. The expression of the TLR4/MyD88/NF-kB pathway increased markedly in the placentae of the GDM patients and this difference was more obvious in the maternal section of the placentae and syncytiotrophoblast of villi. Moreover, heightened local IR caused by impaired insulin signaling has been observed in the placentae of GDM compared with the control group. Furthermore, the expression of TLR4 is positively correlated with local IR in placentae and maternal hyperglycemia. The TLR4/MyD88/NF-kB pathway may play a potential role in the development of IR in placentae of GDM.

## Supporting Information

S1 File The basic information of objects and the results of western bolt. The basic characteristic of GDM patients and control pregnant women, and the relative expression of key molecules of TLR4 pathway and insulin pathway.
(SAV)

S2 File The mRNA level of key molecules of TLR4 pathway. The relative expression of key molecules of TLR4 pathway by Real time-PCR.
(XLS)
